# Overexpression of Eimeria tenella Rhoptry Kinase 2 Induces Early Production of Schizonts

**DOI:** 10.1128/spectrum.00137-23

**Published:** 2023-06-01

**Authors:** Adeline Ribeiro E. Silva, Mamadou Amadou Diallo, Alix Sausset, Thomas Robert, Stéphane Bach, Françoise I. Bussière, Fabrice Laurent, Sonia Lacroix-Lamandé, Anne Silvestre

**Affiliations:** a ISP, INRAE, Université de Tours, Nouzilly, France; b Sorbonne Université, CNRS, UMR 8227, Integrative Biology of Marine Models Laboratory (LBI2M), Station Biologique de Roscoff, Roscoff, France; c Sorbonne Université, CNRS, FR 2424, Plateforme de Criblage KISSf (Kinase Inhibitor Specialized Screening Facility), Station Biologique de Roscoff, Roscoff, France; d Centre of Excellence for Pharmaceutical Sciences, North-West University, Potchefstroom, South Africa; Weill Cornell Medicine

**Keywords:** *Eimeria*, Apicomplexa, kinase, rhoptry, ROPK, p38 MAPK

## Abstract

Eimeria tenella is an obligate intracellular parasite responsible for avian coccidiosis. Like other apicomplexan parasites, such as Toxoplasma gondii, cell invasion and intracellular development rely on apical organelle content discharge, named micronemes and rhoptries. Some rhoptry (ROP) kinases (ROPK) are key virulence factors in T. gondii. To date, among the 28 *ropk* genes carried by E. tenella, only two to four were confirmed by proteomic analysis or immunostaining to be expressed at the sporozoite stage. We have previously shown that EtROP1 is implicated in the inhibition of host cell apoptosis by interacting with the cellular p53. This work functionally described the second ROP kinase expressed at the sporozoite stage in E. tenella. EtROP2 is an active kinase that phosphorylates cell substrates of approximately 50 kDa. Its overexpression leads to the shortening of the prepatent period and to the early development of first-generation schizonts. Conduction of RNA sequencing analysis and reverse transcriptase quantitative PCR (RT-qPCR) on the host cell allowed us to identify the mitogen-activated protein kinase (MAPK) pathway and the transcription factor cFos to be upregulated by EtROP2. We also showed by immunofluorescence assay that the active kinase EtROP2 is implicated in the p38 MAPK pathway activation. We established here that EtROP2 activates the p38 MAPK pathway through a direct or indirect phosphorylation, leading to the overexpression of the master transcription factor cFos known to be implicated in E. tenella development.

**IMPORTANCE** Rhoptries are specialized secretory organelles found in zoite stages of apicomplexan parasites. In addition to well-conserved rhoptry neck proteins, their protein consists mostly of kinase proteins, highly divergent from eukaryotic kinases. Some of those kinases are described as major virulence factors in Toxoplasma gondii, secreted into the host cell to hijack signaling pathways. Most of those kinases remain to be characterized in *Eimeria tenella*. Deciphering their cellular function is a prerequisite to supporting their relevance as a druggable target in development of new means of *Eimeria tenella* control. Secreted divergent kinases that interact with host cell partners to modulate pathways are good candidates, as they coevolve with their host targets to ensure their function within the host and are less prone to mutations that would lead to drug resistance. The absence of any orthologous kinase in host cells makes these parasite kinases a promising drug target candidate.

## INTRODUCTION

Eimeria tenella is an obligate intracellular parasite which belongs to the Apicomplexa phylum. This phylum includes parasites responsible for human and animal diseases such as toxoplasmosis, malaria, cryptosporidiosis, and coccidiosis (1). *Eimeria* spp. are host specific, and seven species (and possibly three cryptic species [2]) are responsible for avian coccidiosis (3). The cost of this worldwide disease is estimated to be of £10.4 billion (calculated at 2016 prices) (4). E. tenella is one of the most virulent species affecting chickens it invades cecal epithelial cells and causes lesions associated with hemorrhagic diarrhea that can lead to death (5, 6). Cell invasion by apicomplexan parasites involves sequential secretion of specialized organelles (7, 8). Micronemal protein (MIC) secretion allows gliding motility and parasite attachment (9, 10) and is followed by rhoptry secretion. Rhoptry neck protein (RON) proteins (located in the neck of rhoptries) participate, in association with MICs, in the gliding motility and the elaboration of the moving junction (11–13) that contributes to propel the parasite into the host cell (14). Rhoptry (ROP) proteins, located in the bulb of rhoptries, are then secreted into the host cell. Some ROP proteins are directed to the cytoplasm or the nucleus of the cell. ROP kinases are specific proteins encountered exclusively in coccidian parasites such as *Toxoplasma*, *Neospora*, and *Eimeria* (15). They were first characterized in Toxoplasma gondii as proteins allowing parasite development by modulation of host signaling pathways (16, 17).

Although rhoptry kinases are restricted to apicomplexans, phylogenetic analysis showed that most of the 28 rhoptry kinases from E. tenella (18, 19) constitute a specific clade, organized into seven subfamilies (20), based on sequence homology and tandem repeat loci. Recently, we confirmed the dynamic transcription of 27 of these *ropk* genes during the parasite life cycle (21, 22). No correlation could be found between subfamilies and stage transcription (21). Three distinct transcription profiles were described: (i) with a constant and low expression across the parasite life cycle, (ii) with higher expression in the extracellular stages, and (iii) with higher expression in the intracellular stages. Among E. tenella putative ROP kinases (ROPKs), two and seven proteins were previously detected at the sporozoite and the merozoite stages, respectively (23–25). EtROP1 is the first kinase that has been functionally characterized in E. tenella (26). Identified at the sporozoite stage, EtROP1 is associated with host cell apoptosis inhibition and with cell cycle arrest in G_0_/G_1_, by phosphorylating the p53/p21 cellular pathway (26).

These new insights prompted us to study the second ROPK identified at the infectious stage of the parasite (25) and predicted to possess a fully conserved catalytic domain (CD) (19, 20). We report here the characterization of EtROP2, coded by the locus ETH_00027700. EtROP2 is a rhoptry bulb kinase that phosphorylates one or several cellular substrates of approximately 50 kDa. We determined the *in vivo* phenotype of an overexpressing EtROP2 strain, and we deciphered the cellular pathways modified/activated by EtROP2 *in vitro*.

## RESULTS

### *Etrop2* sequence analysis.

**FIG 1:**
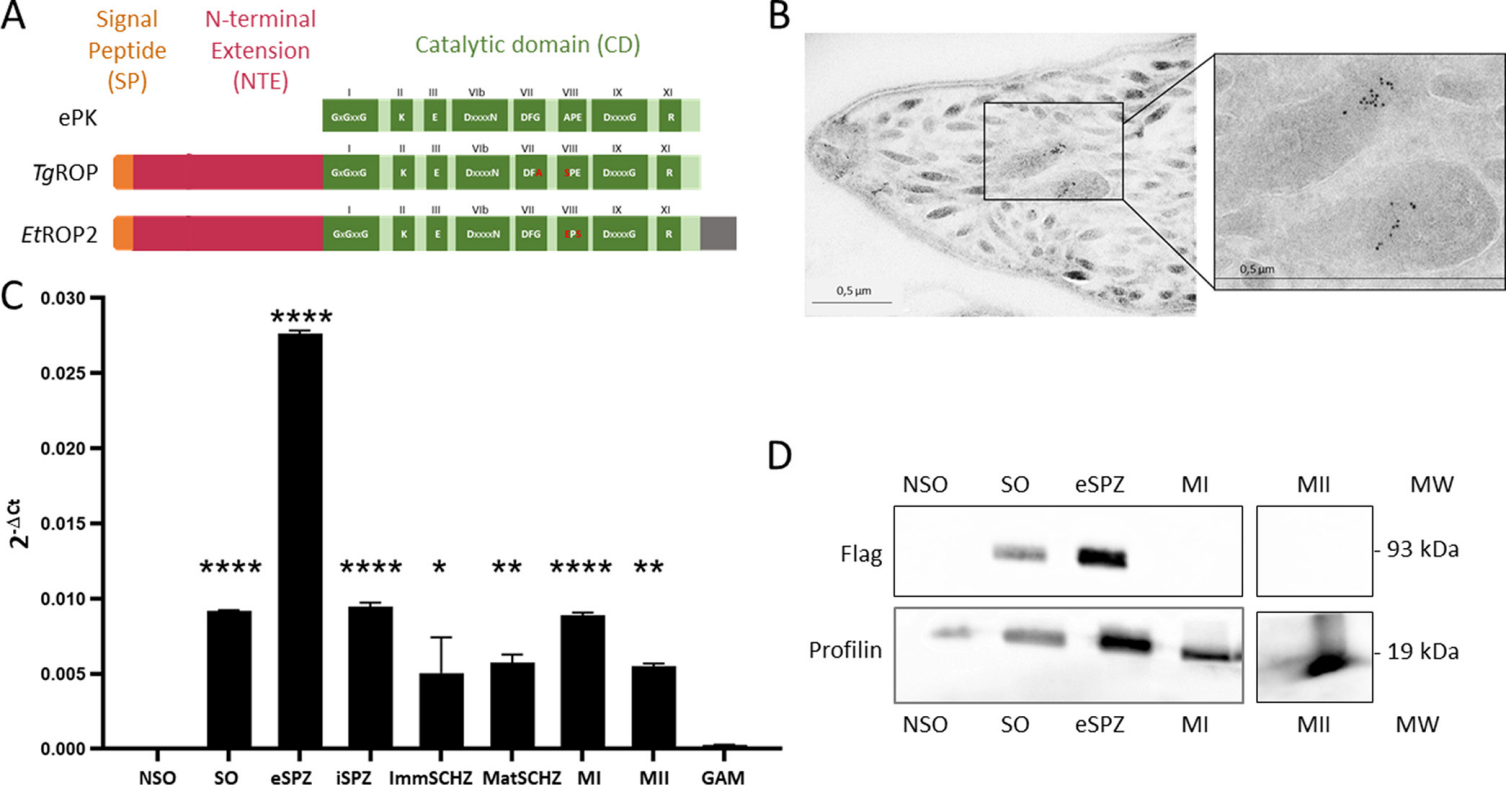
Rhoptry kinase EtROP2 is highly expressed during the extracellular sporozoite stage of the E. tenella life cycle. (A) *EtROP*2 sequence analysis. The locus ETH_00027700 is annotated as a rhoptry kinase family protein. It contains a catalytic domain (CD from Val323 to Phe665) of a serine-threonine kinase, with highly conserved amino acids distributed in 11 subdomains (Roman numerals). BLAST analysis with Toxoplasma gondii identified TGME49_270920 to be more closely related to EtROP2. Polymorphism between T. gondii rhoptry kinase, EtROP2, and eukaryotic kinase (ePK) gene products are indicated by red amino acids. Both parasite kinases diverge from ePK by their N-terminal extension (NTE) containing a signal peptide (SP) and by the C-terminal extension (Cter) for EtROP2. (B) The immunogold revelation of EtROP2-tagged Flag shows that EtROP2 is localized in the rhoptry bulb compartment, at the anterior pole of the parasite. (C) *Etrop2* transcript expression was measured by RT-qPCR in all developmental stages: nonsporulated oocysts (NSO), sporulated oocysts (SO), extracellular sporozoites (eSPZ), intracellular sporozoites (iSPZ), immature schizonts (ImmSCHZ), mature schizonts (MatSCHZ), first-generation merozoites (MI), second-generation merozoites (MII), and gametes (GAM). Results are expressed as relative gene expression using *Etactin* and *Et18S* as housekeeping genes. Ordinary one-way analysis of variance (ANOVA) was performed using Prism v8 software (GraphPad Software, San Diego, CA, USA), and Tukey’s test was used to correct for multiple comparisons against the NSO stage. *, *P* < 0.05; **, *P* < 0.01; ****, *P* < 0.0001. (D) Western blot analysis performed on five extracellular parasite stages. The recombinant EtROP2 was detected by an anti-Flag antibody. An anti-profilin antibody was used to normalize the protein load.

ToxoDB was queried to analyze the ETH_00027700 locus corresponding to the *Etrop2* gene. The protein-primary sequence deduced was used to analyze the EtROP2 protein, which was annotated as a ROP kinase. ETH_00027700 is described as a ROP25 putative kinase; however, there is no clear ortholog in *Toxoplasma* (neither TgROP25 nor TgROP2). EtROP2 consists of a 671-amino acid (aa) protein with a predicted molecular weight of 71 kDa. EtROP2 is predicted to possess a putative serine/threonine kinase domain at residues 323 to 665, with the conservation of 11 subdomains and most key residues that are essential for the canonical kinase activity (27). Despite the lack of conserved alanine and glutamic acid residues in subdomain VIII, these bioinformatic predictions suggested that EtROP2 was an active kinase (21). The analysis indicated an N-terminal extension (NTE), also found in other ROPKs (20), containing a signal peptide (SP) with a cleavage site between residues 16 and 17. A short C-terminal extension (Cter) was predicted in EtROP2 at residues 666 to 671. A Cter extension can be found in some ROPKs and some eukaryotic protein kinases (ePK), and their size can vary from a few residues (EtROP2) to many, as for EtROP1 (26) (Fig. 1A).

### EtROP2 is a rhoptry protein.

To determine the subcellular localization of EtROP2, we constructed a recombinant strain of the E. tenella parasite. The *Etrop2* gene was fused at its 3′ end with a yellow fluorescent protein (YFP) epitope and a Flag tag and was expressed under its endogenous promoter. After several rounds of *in vivo* propagation and fluorescence-activated cell sorter (FACS) selection of recombinant parasites, transmission electron microscopy (TEM) was run on recombinant sporozoites. An immunogold analysis labeling the Flag-tagged EtROP2 protein confirmed its localization in the rhoptry compartment and, more specifically, in the bulb of the rhoptries (Fig. 1B). Both the sequence analysis and the localization demonstrated that *Etrop2* encodes a rhoptry protein.

### EtROP2 expression along the E. tenella life cycle.

We next investigated the expression of EtROP2 at different parasite stages during the E. tenella life cycle by reverse transcriptase quantitative PCR (RT-qPCR). Nine purified parasite stages were investigated: nonsporulated oocysts (NSO), sporulated oocysts (SO), extracellular sporozoites (eSPZ), intracellular sporozoites (iSPZ), immature schizonts (ImmSCHZ), mature schizonts (MatSCHZ), first-generation merozoites (MI), second-generation merozoites (MII), and gametes (GAM). As shown in Fig. 1C, there is significantly higher expression of *Etrop2* in the sporozoites, no expression at the NSO and GAM stages in agreement with previous transcriptomic data (28, 29), and moderate expression in all other explored stages. In the absence of synchronous development of E. tenella, we cannot rule out that ImmSCHZ and MatSCHZ can be partly contaminated by iSPZ and ImmSCHZ, respectively, resulting in slightly overestimated *Etrop2* transcription in those stages. To highlight a possible regulation of *Etrop2* transcripts, we investigated the protein expression on NSO, SO, eSPZ, MI, and MII. A Western blot analysis using an anti-Flag antibody confirmed that EtROP2 is expressed in the sporozoites and in sporulated oocysts that contain eight mature sporozoites (Fig. 1D). This expression is restricted to the first infectious stage of the parasite, and its localization in the bulb of rhoptries suggests a role of EtROP2 during the intracellular development of the parasite.

### EtROP2 is an active kinase.

**FIG 2:**
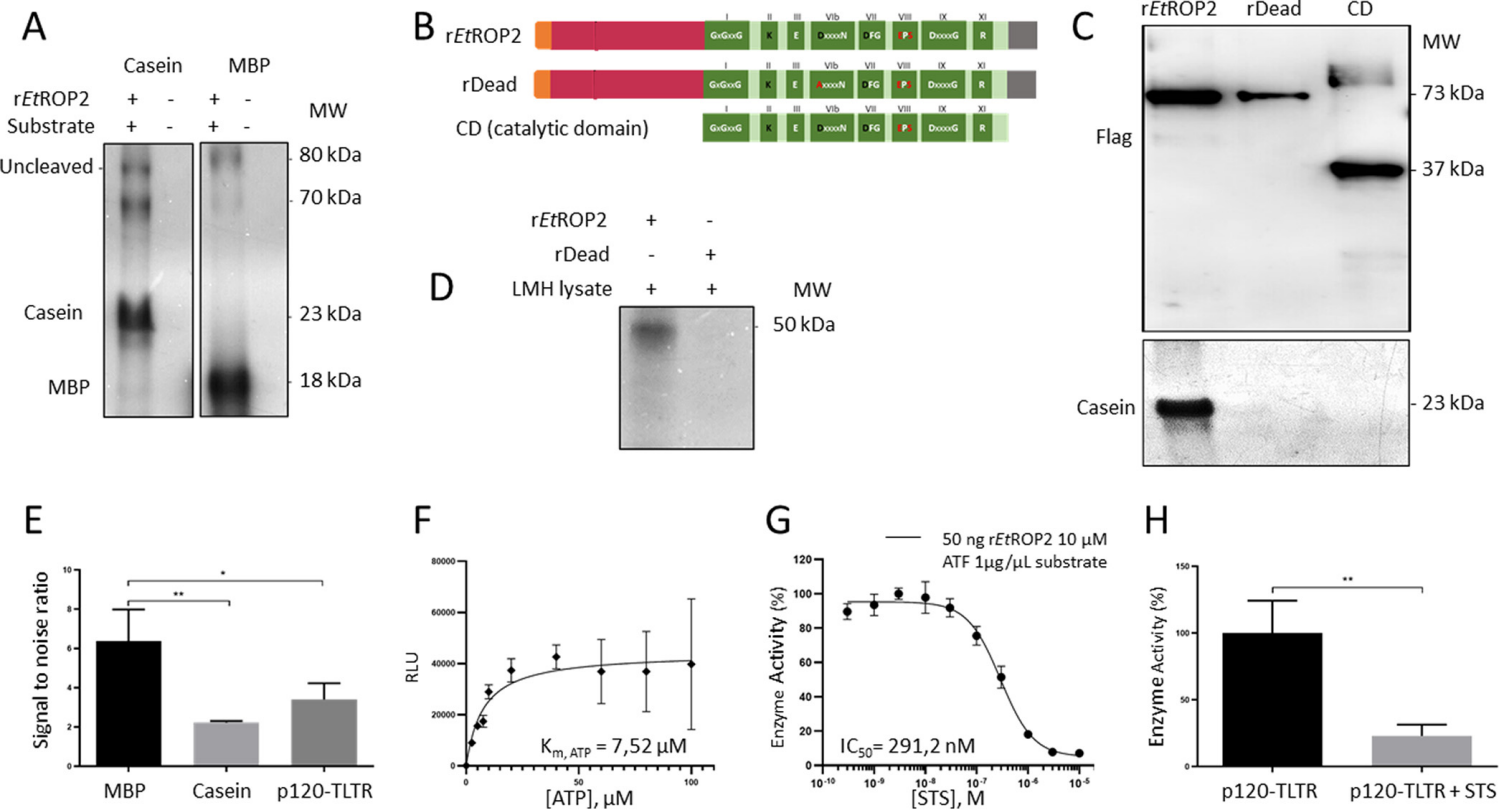
EtROP2 is an active kinase. (A to H) All the recombinant proteins used were tagged in their C-terminal extension with a Flag (A to D) or with glutathione *S*-transferase (GST) (E to H): wild-type form rEtROP2 (full-length, from Met1 to Glu671), inactive form rDead, in which the aspartate from KDD motif was mutated into alanine D513A, and the truncated form, with the N-terminal extension deleted from (Met261 to Glu671), encompassing the catalytic domain (CD). (A) SDS-PAGE autoradiography of a kinase assay performed on rEtROP2 in the presence or absence of α-casein or MBP substrate. (B) Scheme of recombinant proteins. (C) Western blot analysis performed on the recombinant forms of the kinase, rEtROP2, rDead, and CD. Recombinant proteins were detected with an anti-Flag antibody (top panel). SDS-PAGE autoradiography of a kinase assay was performed on all recombinant forms in the presence of α-casein (bottom panel). (D) SDS-PAGE autoradiography of a kinase assay performed on the rEtROP2 and rDead forms of the kinase, incubated with LMH cell lysate. (E) Identification of peptide phosphorylation substrates of EtROP2-GST. *In vitro* kinase activity of EtROP2-GST against peptides corresponding to MBP (black), casein (light gray), and p120-TLTR (dark gray) using 50 ng of EtROP2-GST and 1 μg of peptide substrates MBP, casein, or p120-TLTR. Kinase assays were performed in triplicates. Signal-to-noise ratios were calculated by dividing each luminescence value with that obtained without protein or peptide substrate (mean ± standard deviation [SD]). Ordinary one-way ANOVA was performed using GraphPad Prism, and Tukey’s test was used to correct for multiple comparisons *, *P* < 0.05; **, *P* < 0.01. (F) Determination of the *K_m_*_, ATP_ of EtROP2-GST/p120-TLTR Shown as a Michaelis-Menten plot of EtROP2-GST/p120-TLTR kinase activity measured by increasing the concentration of ATP (0 to 100 μM). In this experiment, 50 ng rEtROP2-GST and 1 μg p120-TLTR per assay were used. Kinase assays were performed in triplicates. The *K_m_*_, ATP_ value, which represents the ATP concentration required to reach the half-maximal kinase activity, was obtained using GraphPad Prism. RLU, relative light unit. (G) Determination of the IC_50_ value of the broad-spectrum inhibitor of protein kinase, staurosporine (STS), against EtROP2-GST. EtROP2-GST was assayed in the presence of increasing concentrations of STS with p120-TLTR as a substrate. Kinase activities are expressed in percentage of maximal kinase activity, i.e., measured in the absence of inhibitor. Mean percentages are reported ± SD. Kinase assays were performed in quadruplicates. The IC_50_ value was determined using GraphPad Prism. (H) Inhibition of the kinase activity of EtROP2-GST/p120-TLTR. The percentage of activity was measured for EtROP2-GST/p120-TLTR in the absence (black) or presence of 1 μM STS (gray). Results are expressed as the percentage of maximal kinase activity, i.e., measured in the absence of inhibitor. Mean percentages are reported ± SD. Kinase assays were performed in triplicates. Student’s *t* test was performed using GraphPad Prism; **, *P* < 0.01.

To characterize the enzymatic activity of EtROP2, three recombinant forms of the kinase were produced in HEK 293T (human embryonic kidney) cells for their propensity for transfection. The wild-type EtROP2 (rEtROP2), the Dead version (D513A mutation of the HRD from the catalytic domain, rDead), and a truncated form, corresponding to the catalytic domain (CD) alone were cloned in fusion with a Flag tag in the Cter in a eukaryotic expression vector (pcDNA). We analyzed the enzymatic activity of the rEtROP2, rDead, and the CD using [γ-^32^P] ATP. Three major phosphorylation signals were obtained (Fig. 2A): two at approximately 70 to 80 kDa, which could correspond, respectively, to the autophosphorylation signals of the matured and uncleaved forms of rEtROP2. Another major signal was obtained and demonstrated that rEtROP2 can phosphorylate the heterologous substrates casein and myelin basic protein (MBP). However, the catalytic domain alone and the Dead version of the kinase were not able to phosphorylate the casein (Fig. 2B and C). These results confirm that the phosphorylation signals obtained are specific to the kinase activity and that the N-terminal extension (NTE) seems to be necessary for the enzymatic activity of EtROP2 as demonstrated for EtROP1 (26). The *in vitro* kinase assay run on LMH (primary epithelial cell line of avian hepatocellular carcinoma) cell lysate indicated that rEtROP2 can phosphorylate host cell proteins of 50 kDa, although we cannot rule out a possible degradation of rEtROP2. The absence of signal with the rDead confirmed that the signal was specific to the kinase activity (Fig. 2D).

### Identification of a peptide substrate for EtROP2 kinase.

In order to detect the enzymatic activity of EtROP2, we performed a bioluminescence-based assay that quantifies the amount of ADP produced during the kinase reaction (30). We first tested the kinase activity on two synthetic peptides corresponding to the MBP and the casein substrates that we previously tested by radioactivity kinase assay (Fig. 2E). We also tested the capacity of EtROP2 to phosphorylate a peptide substrate derived from the p120-catenin: the p120-TLTR (DANPLMANGTLTRRHQNGRF) (31), a nonspecific substrate available in the synthetic peptide library. We confirmed the capacity of EtROP2 to phosphorylate peptides corresponding to both commercial protein substrates, casein and MBP, and we also demonstrated that EtROP2 can phosphorylate the p120-TLTR peptide (Fig. 2E). As p120-TLTR presents a higher luminescent signal than the casein, and as the MBP is composed of multiple peptide sequences, we selected p120-TLTR as a privileged substrate for the enzymatic experiments described in this study.

### Determination of the *K_m_*_, ATP_.

Using the optimal concentrations (50 ng rEtROP2-GST and 1 μg p120-TLTR), we next determined the ATP concentration required to reach the half-maximal kinase activity (*K_m_*_, ATP_) of rEtROP2-GST/p120-TLTR using a homogeneous luminescent ADP detection assay to measure kinase activity by quantifying the amount of ADP produced during a kinase reaction (ADP-Glo assay kit, Promega, Madison, WI). In this assay, we tested increasing concentrations of ATP: from 0 to 100 μM. The *K_m_*_, ATP_ measured was about 7.52 μM. This is the first *K_m_*_, ATP_ value determined experimentally for an E. tenella ROPK (Fig. 2F), and this is in the same range as the *K_m_*_, ATP_ determined for TgROP18 (3 μM) in T. gondii (32).

### Inhibition of the EtROP2 activity.

In the absence of EtROP2-specific inhibitors, we tested the effect of the broad-spectrum kinase inhibitor staurosporine (STS). As shown on Fig. 2G, the indolocarbazole compound STS inhibits rEtROP2-GST/p120-TLTR with a half-maximal inhibitory concentration (IC_50_) of 291.2 nM. This value stays in the range of the IC_50_ obtained for other kinases (33, 34): e.g., TgROP18 was shown to be inhibited by ATP competitive compounds with IC_50_ values between 170 and 300 nM (32). As shown in Fig. 2H, the kinase activity of rEtROP2-GST with p120-TLTR as a substrate is inhibited at 75% in the presence of 1 μM STS.

### The overexpression of EtROP2 reduces the prepatent period.

**FIG 3:**
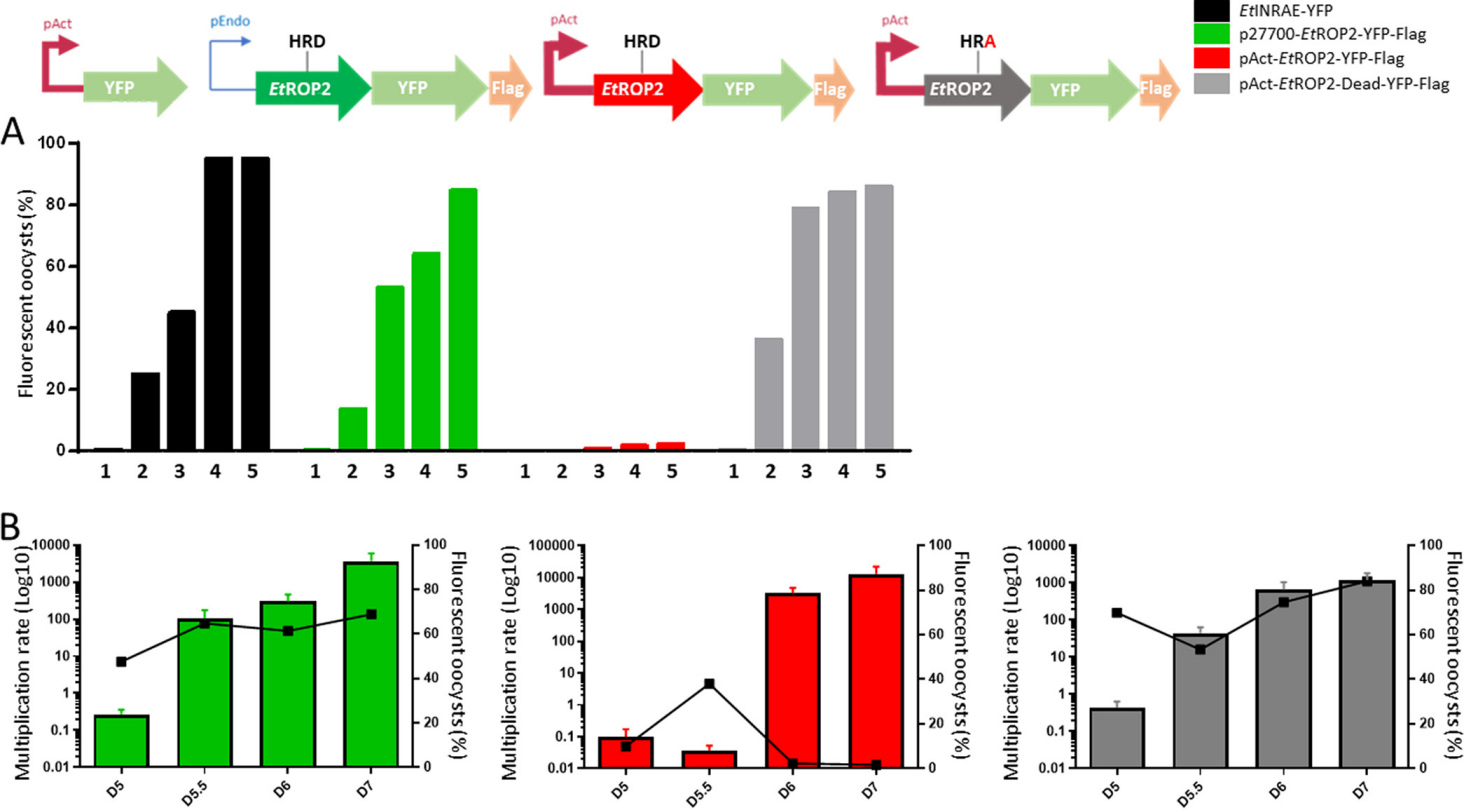
Recombinant parasite production. (A) The wild-type strain and three recombinant strains were propagated through five passages in chickens (P1 to P5) in order to increase the percentage of fluorescent parasites in the recombinant populations. Results are expressed in percentage of fluorescence. (B) Monitoring of the daily multiplication rate (i.e., daily parasites produced/parasites inoculated) and percentage of fluorescence *in vivo* at days 5, 5.5, 6, and 7 postinfection. The daily multiplication rate is expressed in log_10_, and fluorescence is expressed in percentage. Mean daily multiplication rates are reported ± SD. Strains are represented as follows: WT EtINRAE-YFP, black; p27700-EtROP2-YFP-Flag, green; pAct-EtROP2-YFP-Flag, red and pAct-EtROP2-Dead-YFP-Flag, gray.

To better understand the function of EtROP2, we generated several recombinant strains, modified to study the level of expression and activity of the kinase EtROP2. In the absence of an efficient protocol to invalidate genes in E. tenella, we compared the phenotype of a wild-type (WT) strain, EtINRAE-YFP, to the phenotypes of three recombinant strains: (i) the p27700-EtROP2-YFP-Flag (p27700) strain, which induces a slight increase of EtROP2 expression due to the multi-insertion copy of the tagged EtROP2, (ii) the pAct-EtROP2-YFP-Flag (pAct) strain, which is associated with an overexpression of *Etrop2* under the actin promoter control, and (iii) the pAct-EtROP2-Dead-YFP-Flag (Dead) strain, which overexpresses the Dead version of the kinase (D513A mutated in the HRD motif from the catalytic domain) under the actin promoter control. Strains were successively propagated in chickens, and using FACS selection, stabilized after five passages, increasing the percentage of recombinant parasites up to 85 to 90% (Fig. 3A), except for the pAct strain. For this strain, at 7 days postinfection (dpi) we collected mainly WT oocysts; no increase in recombinant parasites was observed. To go further, we investigated the possibility of early production of the recombinant parasites as was shown for T. gondii with the overexpression of TgROP18 (35). We thus measured the multiplication rate and the percentage of recombinant parasites at days 5, 5.5, 6, and 7 postinfection for each recombinant strain. P27700 and Dead strains exhibit the same profiles, with a multiplication rate higher at 7 dpi and a percentage of fluorescence that remains constant. For the pAct strain, we also observed a multiplication rate higher at 7 dpi; however, the percentage of fluorescence was considerably higher at 5.5 dpi and dramatically decreased at 6 and 7 dpi (Fig. 3B). These results show that the overexpression of the active kinase induces early production of recombinant parasites, while WT parasites generated within *in vivo* passage appear at 6 to 7 dpi.

Selection of oocysts from the pAct strain at 5.5 dpi allowed the stabilization of the strain after five passages, with a fluorescent rate reaching 90%. To confirm the property of our three recombinant strains, the localization of EtROP2 was confirmed by immunofluorescence assay at the apical tip and by TEM in rhoptries (see Fig. S1A in the supplemental material). Transcript expression was also compared using *Etrop2* and *yfp* primer amplification on sporulated oocyst RNA (Fig. S1B). Surprisingly, the results showed that the overexpression of *Etrop2* and *yfp* genes was achieved only in the pAct strain. Protein detection with anti-Flag antibody confirmed the overexpression only for the pAct strain. As 5′-untranscribed-region (UTR) and promoter sequences used for sporozoite transfection were checked, these results suggest a possible deleterious effect of the overexpression of dead EtROP2.

### Monitoring of parasite stages during the life cycle.

**FIG 4:**
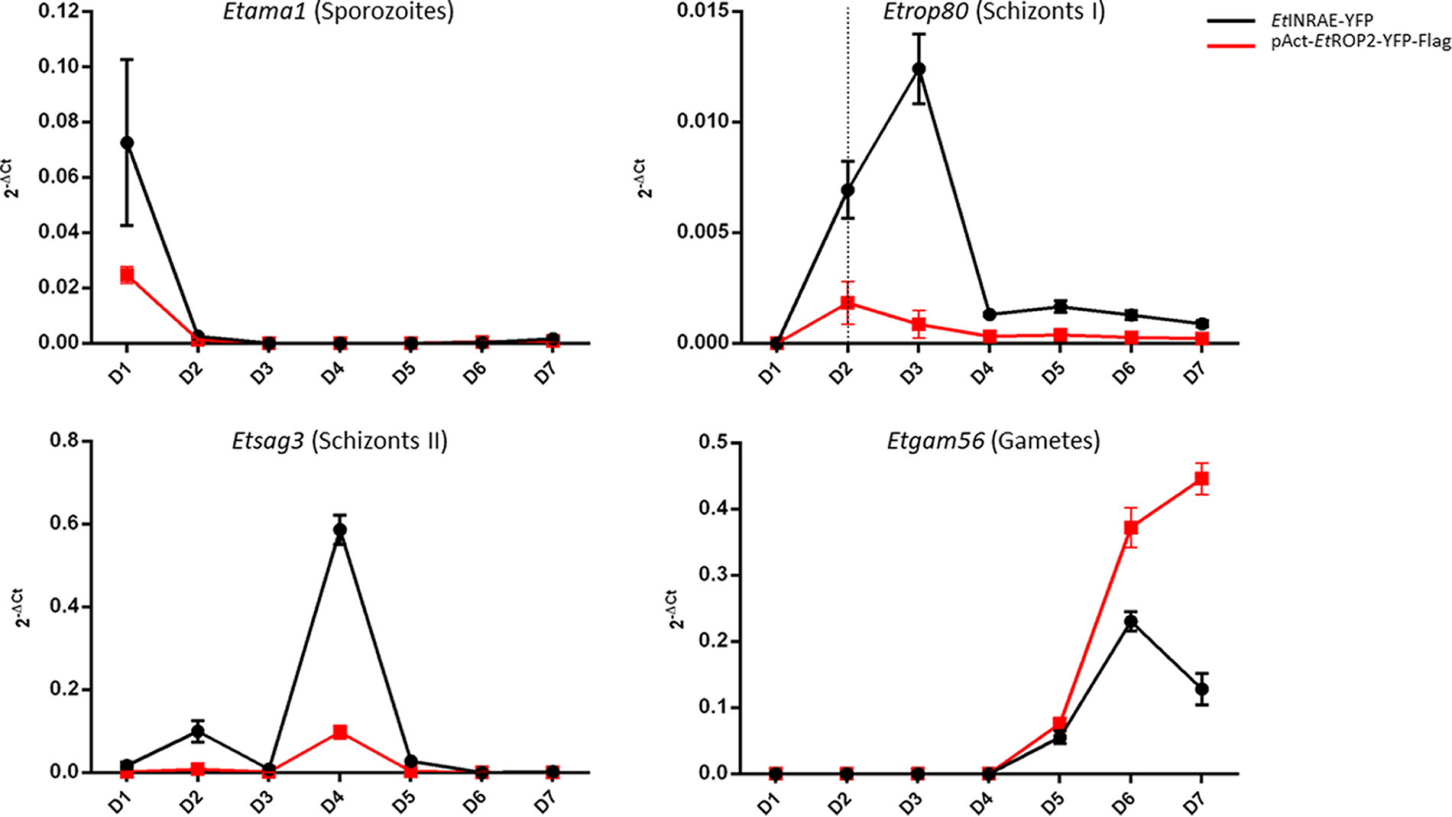
Evaluation of WT and pAct E. tenella development *in vivo*. Expression of *Etama1*, a gene specific to the sporozoite stage; *Etrop80*, first schizont and merozoite stages; *Etsag3*, second schizont and merozoite stages; *Etgam56*, gametes and nonsporulated oocyst stages. Transcripts were detected in infected ceca with the WT (black) or pAct-EtROP2-YFP-Flag (red) strain from 1 to 7 dpi. The dotted vertical line represents the time with the maximum *Etrop80* transcripts observed for the pAct-EtROP2-YFP-Flag strain. Each measure was realized in triplicates on two biological samples. Results are expressed in 2^–Δ^*^CT^* ± SD. Normalization was obtained with *Etactin* and *Et18S* housekeeping genes.

The shortening of the prepatent period observed *in vivo* with the pAct strain (overexpressing EtROP2) can be explained either by the global shortening of developmental stages or by the absence of one specific stage. In some vaccine strains, which also have a prepatent period of 5 days instead of 7 days for WT strains, one merogony is absent. We monitored the daily production of parasite stages in chickens infected with the WT or the pAct strain using four stage-specific genes. This qualitative method allows the detection of stage emergence in ceca but not quantitatively. Sporozoites are detected at 1 dpi for the WT and pAct strains (Fig. 4). First schizonts and merozoites are detected at 2 dpi for the pAct strain, while they are mainly detected at 2 to 3 dpi for the WT strain. No difference was observed between strains at the second merozoite and gamete stages. Histological analysis of infected chickens from day 1 to day 7 also confirmed the early production of parasites with the codetection of immature and mature schizonts 3 dpi in pAct-infected chickens, whereas only immature schizonts are visible in WT strain at this time (Fig. S2).

### EtROP2 overexpression induces early production of schizonts.

**FIG 5:**
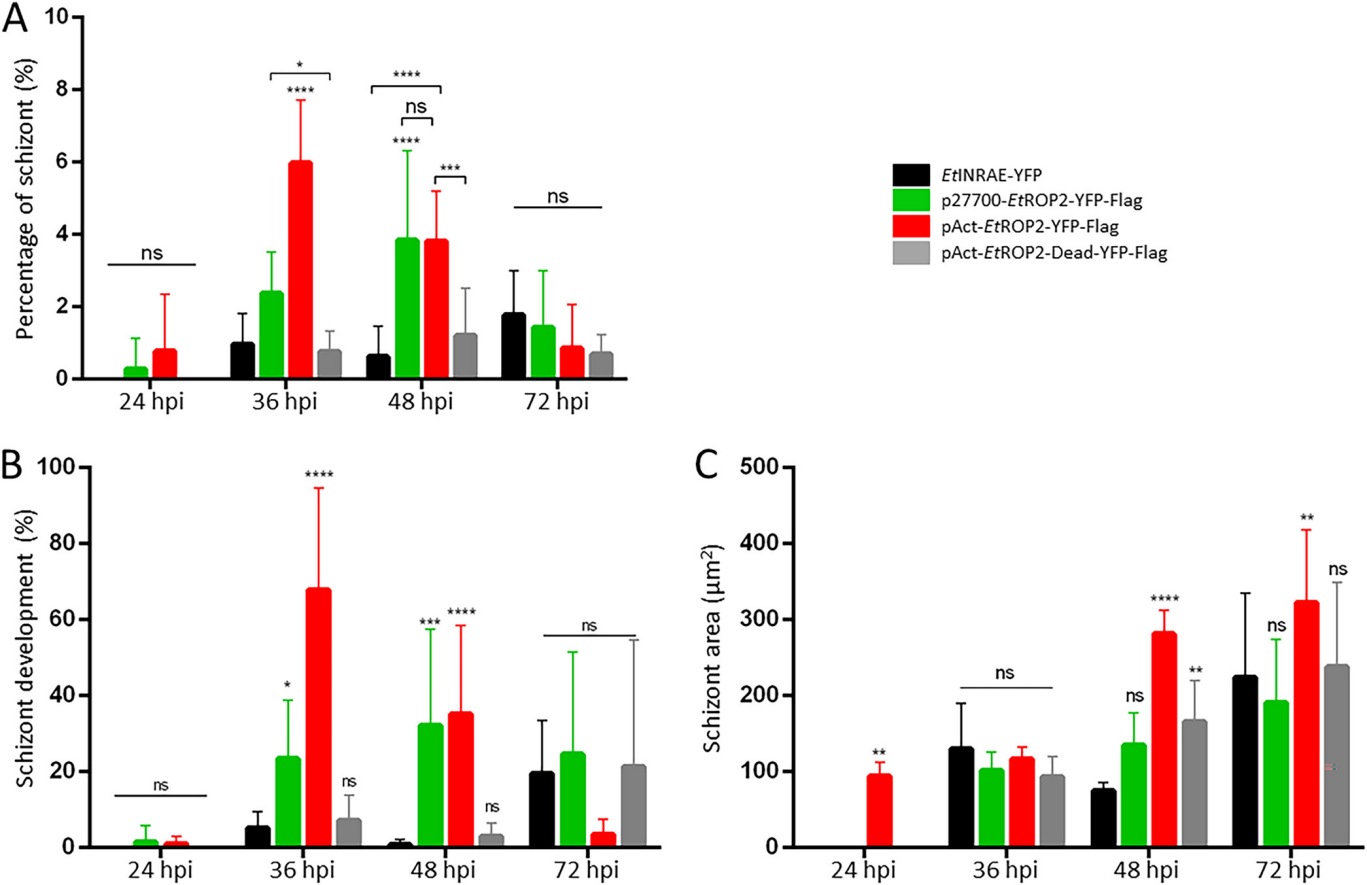
Development assay. Development was measured on CLEC213 infected with the same strains as for the invasion assay at 24, 36, 48, and 72 hpi. (A) Percentage of schizonts observed for each developmental strain. The percentage of schizonts is higher at 36 hpi for the pAct strain with a mean percentage of 5.98 ± 1.74, compared to 0.97 ± 0.84 for the WT, 2.38 ± 1.14 for p27700, and 0.77 ± 0.56 for the Dead strains. (B) Percentage of invaded sporozoites that developed into schizonts. The percentage is higher at 36 hpi for the pAct strain than for other strains. (C) Mean of the schizont’s area, which is proportional to the MI number and thus to the schizont maturity. It appears that schizonts are larger for the pAct strain at 48 and 72 hpi, suggesting the presence of mature schizonts. Results are expressed as percentage of schizonts ± SD, percentage of development ± SD, and μm^2^ ± SD for the schizont area. WT, black; p27700, green; pAct, red; Dead, gray. Nine replicates were performed in order to cope with biological variability. Ordinary two-way ANOVA was performed using GraphPad Prism, and Tukey’s test was used to correct for multiple comparisons. *, *P* < 0.05; **, *P* < 0.005; ***, *P* < 0.0005; ****, *P* < 0.0001.

Since EtROP2 was strongly expressed at the sporozoite stage, we investigated whether the shortening of the prepatent period could be the result of early events occurring at the schizont I stage, which can be easily obtained *in vitro*. For that, we estimated the percentage of schizont production *in vitro*. The production of first-generation schizonts significantly increased in pAct strain at 36 h postinfection (hpi), compared with the three other strains and at 48 hpi, in comparison to the WT strain (Fig. 5A). The same phenotype was observed for the p27700 strain, with a slight increase at 36 hpi, confirmed at 48 hpi. We evaluated the percentage of schizont development; it means the capacity of invaded parasites to develop into schizonts (ratio between [immature + mature schizont]/intracellular sporozoites). As shown in Fig. 5B, the pAct strain presented a significantly higher schizont development than the three other strains at 36 and 48 hpi. The p27700 strain had the same phenotype as pAct, with higher schizont development at 36 and 48 hpi. Finally, we evaluated the number of MI in mature schizonts. For that, we considered that the number of MI was proportional to the schizont area (36). The pAct strain showed a significantly higher schizont area at 48 hpi (281 μm^2^) and 72 hpi (322 μm^2^) compared to other strains (75 to 165 μm^2^ and 191 to 238 μm^2^, at 48 and 72 hpi, respectively) (Fig. 5C).

We gained evidence that overexpression of the active EtROP2 kinase led to early production of mature schizonts. Those results showed that EtROP2 was implicated in the prolificity of the parasite at the first schizogony. Those results also confirmed that EtROP2 did not modify the invasion process: no difference was observed between the WT and the recombinant strains during invasion (Fig. S3).

### The active EtROP2 induces the p38 MAPK cellular pathway.

**FIG 6:**
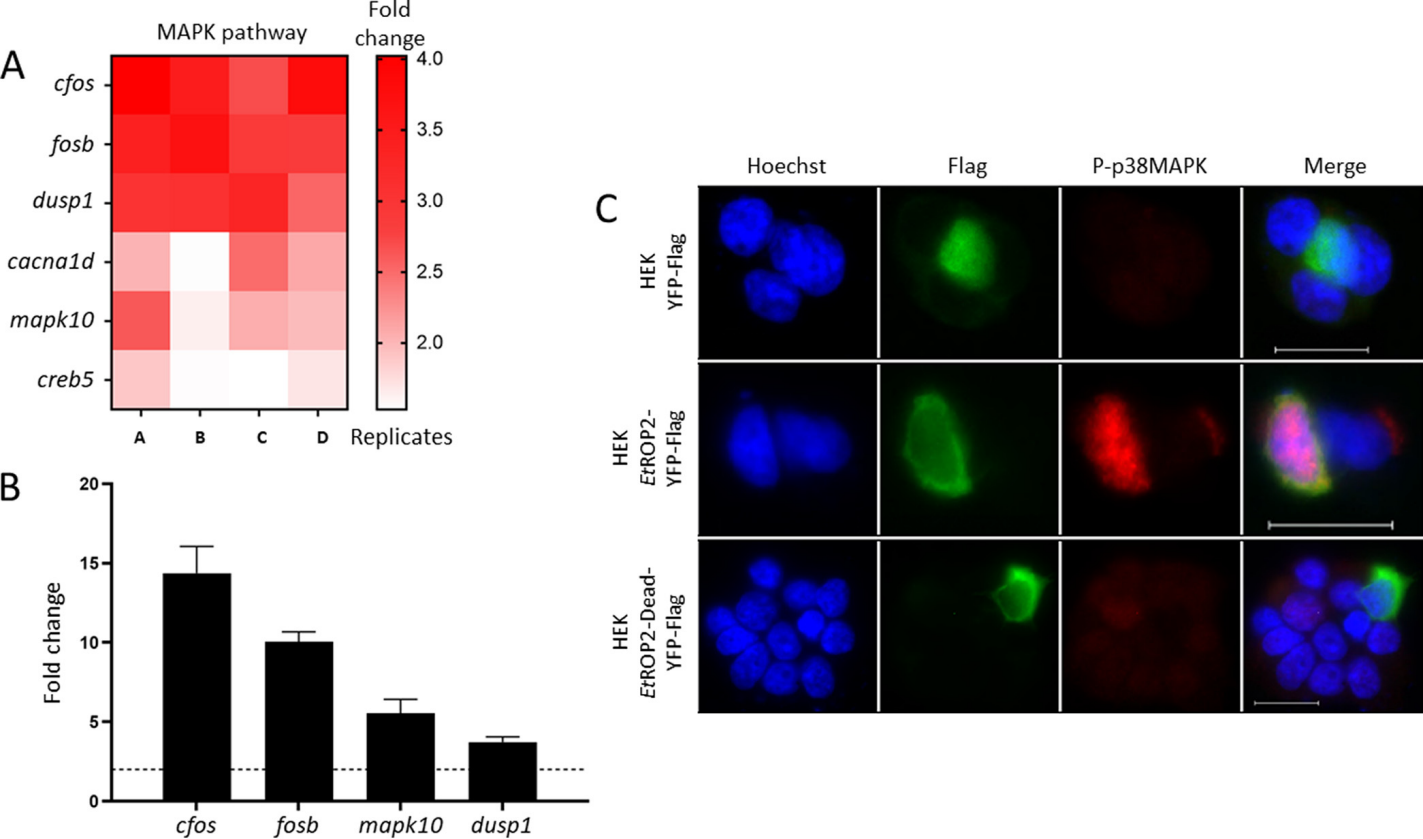
Host transcripts regulated by the active EtROP2. (A) Genes implicated in the MAPK pathway and altered by the expression of the active EtROP2 in transfected CLEC213 cells. Each panel represents four biological replicates. (B) Quantitative RT-PCR expression profile of the MAPK pathway genes. *Cacna1d* expression was not analyzed. Results are expressed in fold change, and threshold modulation of transcripts (≥2) is marked by a horizontal black line. (C) Immunostaining of phospho-p38-MAPK upon transfection of HEK 293T cells by YFP-Flag, EtROP2-YFP-Flag, or EtROP2-Dead-YFP-Flag plasmid. Host cells were stained with rabbit monoclonal anti-phospho-p38-MAPK (Thr180/Tyr182) antibody (red). Recombinant proteins are visualized in green, and Hoechst was used to visualize host nuclei. Bar scale = 20 μm.

To investigate the host genes possibly modified by EtROP2 after cell invasion; transcriptomic analyses were conducted on CLEC213 cells transfected with a control pcDNA-eGFP-Flag or with pcDNA-EtROP2-YFP-Flag plasmid 48 h after transfection. Among the 141 differentially expressed genes (DEG) in EtROP2 transfected cells, 15 were downregulated and 126 were upregulated (Fig. S4, Table S1). Fold changes of >2 were considered significant and were analyzed using the KEGG pathway database: 61 genes presented a significant upregulation (and none were downregulated), revealing an enrichment of 40 pathways. Approximately half of these pathways are related to signaling and a quarter to apoptosis and metabolism (Table S2). Among those pathways, only two were significantly enriched using DAVID v6.8 (expression analysis systematic explorer (EASE) score, ≤0.1) with a *P* value of <0.05: the GnRH and MAPK signaling pathways. MAPK signaling, which is affected during *Eimeria* spp. infections (37–41), is the most enriched pathway, with five upregulated genes. Several mediators of the MAPK signaling pathway are significantly upregulated; among them are cFos and FOSB, two master transcription factors known to be induced by the MAPK pathway, DUSP1, a phosphatase activated by p38 MAPK in order to inactivate the Jun N-terminal protein kinase (JNK) pathway, which protects the cell from apoptosis, CACNA1D, a voltage-dependent Ca^2+^ channel involved in hypermigratory phenotype of T. gondii-infected macrophages, to favor parasite dissemination into the host (42), and the MAPK10 kinase, also named JNK3 (Fig. 6A). Upregulation of those genes was further confirmed by RT-qPCR performed on RNA purified from transfected CLEC213 cells (Fig. 6B).

Immunostaining analyses were also conducted to confirm the activation of the cellular MAPK pathway (Fig. 6C). Only the active EtROP2 led to the phosphorylation of p38 MAPK protein, which induced its nuclear translocation. As MAPK10/JNK3 was also upregulated, we investigated the JNK pathway activation by immunostaining, but neither phosphorylation signal nor nuclear translocation was observed in EtROP2 transfected cells. Our work confirmed that EtROP2 activated the p38 MAPK pathway, leading to cFos upregulation. As cFos is known to be necessary for *Eimeria* sp. development (40), it appears that EtROP2 could be one of the parasite proteins that allows the early *in vivo* development of the parasite by modulating the p38 MAPK cellular pathway.

## DISCUSSION

We identified an active kinase expressed in the rhoptries of E. tenella sporozoites, which affects the early development of the parasite. We previously demonstrated that the *ropk* genes are dynamically expressed during the E. tenella life cycle (21). To date, only EtROP1 has been functionally characterized and associated with parasite survival (26). Other ROPKs, EtROP30 (ETH_000027705), EtROPK-Eten5-A (ETH_00005405), EtROP35 (ETH_000005905), and EtROP1 (ETH_00005190), have been only partially studied for their immunogenicity (22, 43–45). ROPK expression is still poorly described in E. tenella, with only two ROPKs identified in the sporozoite proteome, which we named EtROP1 and EtROP2 (25), without any clear orthologs in T. gondii, and seven in the merozoite proteome, encoded by ETH_0000075, ETH_0005400, *ETH_0005840*, *EtROP35*, *ETH_00026495*, *ETH_00027695* and ETH_00028765 (23, 24). Some of those ROPKs are predicted to be catalytically active based on the conservation of the canonical kinase domain of the ePKs (20, 46). The expression of *Etrop2* at the infectious stage of the parasite (21) and the conservation of residues needed for the catalytic activity prompted us to focus on this ROPK. During this study, we showed that (i) EtROP2 possesses characteristic kinase features and the conserved organization in 11 subdomains (except the glutamic acid absent from subdomain VIII) and (ii) EtROP2 phosphorylates cellular proteins and its NTE appears to be essential for its kinase activity. Following our previous study of EtROP1 (26), our results confirmed the expression of a second catalytically active kinase in the rhoptries of sporozoites.

We observed that overexpression of the active kinase EtROP2 accelerates parasite development *in vitro* and *in vivo*, suggesting the involvement of EtROP2 in cellular pathway modulation, as was shown for the key virulence factor TgROP18 (35). We demonstrated that EtROP2 triggers the activation of the host p38 MAPK pathway and cFos upregulation. As we could not identify any p38 residues phosphorylated by EtROP2, we assumed that the activation of the cellular p38 MAPK pathway by EtROP2 may be indirect and involves other cellular partners. To support this hypothesis, a BLAST analysis of EtROP2 against the Gallus gallus proteome identified a MAPKKKK (XP_040511054) sharing 32% (E value, 1 × 10e-10) protein identity with EtROP2. We can hypothesize that EtROP2 may act as a functional mimic of a host MAPKKKK, activating the MAPK cascade.

The p38 MAPK pathway is activated by a wide range of cellular stresses, including UV irradiation, heat shock, osmotic stress, calcium signal (47), and proinflammatory cytokines (48), which seems to be a hallmark of viral (49), bacterial (50), and Apicomplexa parasite infection (18, 40, 51). In T. gondii, the molecular mechanism was deciphered, and four parasite-secreted effectors have been found to be involved in regulation of the p38 MAPK pathway: TgGRA24 (52), TgMIC1, TgMIC4 (53), and TgROP18 (54). Although the molecular mechanism seems to be different in E. tenella, the p38 MAPK cellular pathway may be a good target for parasite control (55, 56). We previously demonstrated that inhibition of the p38 MAPK pathway resulted in a highly significant inhibition of cell invasion by E. tenella (90%) (37), which is due to the effect on the parasite (70%) and also on the host cell (30%). p38 MAPK activation leads to the induction of cFos, which forms AP-1 complex (with FosB, Fra1, and Fra2) and binds in the regulatory regions of many genes (57) to regulate differentiation, proliferation, apoptosis, and immune response in a wide range of cell types (58). Despite cFos properties in the modulation of inflammatory gene expression, it was also shown to be involved in *Eimeria* development (40).

In the context of new therapeutic developments, EtROP2 can be considered a promising new drug target. Its inhibition may be efficient at controlling/regulating first schizogony, an early step of E. tenella infection (controlling the number of MI produced). Some ROPK inhibitors have already been identified with success to reduce parasite infection *in vivo* (32). There are advantages to targeting ROPK: (i) ROPK has parasite-specific kinases with no host equivalent, supporting a high therapeutic index; (ii) ROPK consists of a highly similar kinase family, so low selectivity of the inhibitor may increase treatment efficacy and reduce the risk of resistance development (even with a possible functional redundancy between ROPK). Development of new drugs to control coccidiosis remains a challenge, considering the social demand to rely on more “natural” control strategies. Their development relies on a better understanding of the ROPK action as virulence factors: direct manner, through their kinase activity (cell cycle arrest by EtROP1), or indirect, through their kinase folding (apoptosis inhibition by EtROP1) (26), which is a necessary process to select the appropriate inhibitors.

**Conclusion.** We characterized the second ROPK expressed in E. tenella and named it EtROP2. We showed that EtROP2 is an active kinase, localized in rhoptries of sporozoites, the first infectious stage of the parasite. We showed the direct effect of the expression of EtROP2 on the first schizogony development, which could be explained by the cellular p38 MAPK activation. Collectively our results support the importance of EtROP2 in the E. tenella life cycle and show that it constitutes a new target for the development of highly specific therapeutics against coccidiosis.

## MATERIALS AND METHODS

### Ethics statements.

All animal experiments were performed in the Infectiology of Farm, Model, and Wildlife Animals Facility (PFIE, Centre INRAE Val De Loire: https://doi.org/10.15454/1.5572352821559333E12, accessed on 25 July 2021; member of the National Infrastructure EMERG’IN). Experimental protocols were designed in compliance with French law (2010/63/EU, 2010; rural code, 2018; decree no. 2013-118, 2013) concerning the use of laboratory animals. Care and euthanasia of animals were conducted according to the national ethical guidelines and approved by the local ethics committee for animal experimentation (Comité d’Ethique en Expérimentation Animale Val de Loire, CEA VdL no. 19): APAFIS no. 25884. The authors are committed to the principles of the 3 Rs: reduction, refinement, and replacement of experimental animals.

### Cell culture and parasites.

Human embryonic kidney cells (HEK 293T) were cultured in Dulbecco’s modified Eagle’s medium (DMEM) containing 10% fetal calf serum (Gibco-Invitrogen, Carlsbad, CA, USA), 2-mM l-glutamine, 50-U/mL penicillin, and 50-μg/mL streptomycin at 37°C under 5% CO_2_. Avian epithelial cell lines LMH (liver epithelial cells) and CLEC213 (chicken epithelial cells) (59) were culture in William’s medium and DMEM (DMEM/F-12), respectively, containing 10% fetal calf serum (Gibco-Invitrogen), 50-U/mL penicillin, and 50-μg/mL streptomycin at 41°C under 5% CO_2_. Madin-Darby bovine kidney cells (MDBK), kindly provided by F. Tomley, were cultured in Ham’s F12 medium (Lonza) containing 10% fetal calf serum (Gibco-Invitrogen), 2-mM l-glutamine, 50-U/mL penicillin, and 50-μg/mL streptomycin at 37°C under 5% CO_2_.

E. tenella INRAE strains expressing (60) or not expression (21) the mCherry fluorescent protein were maintained by serial passages in chickens.

### Oligonucleotides.

E. tenella-specific primers were designed on the basis of nucleotide sequences collected from ToxoDB (http://toxodb.org). Avian-and human-specific primers were designed on the basis of nucleotide sequences collected from NCBI (https://www.ncbi.nlm.nih.gov/). All primers were purchased from Eurogentec (Table S3).

### Sequences analysis.

Ortholog analyses were performed using BLAST (http://www.ncbi.nlm.nih.gov/blast/). Proteins were aligned using MultAlin (61). Potential signal peptide cleavage sites were identified with SignalP 3.0 (http://www.cbs.dtu.dk/services/SignalP/) (62). Functional domains were predicted using PROSITE (63). Signaling pathway identification was predicted using the KEGG pathway database (https://www.genome.jp/kegg/pathway.html).

### Plasmid construction.

We produced plasmids expressing *Etrop2* (ETH_00027700) under its wild-type form (WT, full-length, from Met1 to Glu671), inactive form (rDead), in which the aspartate from the HxD motif was mutated into alanine D513A using the Q5 site-directed mutagenesis (NEB corporation, Fond Du Lac, WI, USA, no. E0554S) according to the manufacturer’s recommendations, and the truncated form, with the N-terminal extension deleted, resulting in a fragment (Met261-Glu671) encompassing the catalytic domain only (CD). For all constructions, *Etrop2* was amplified by PCR using the polymerase Phusion high fidelity DNA polymerase (Fermentas Corporation Waltham, MA, USA) and cloned between the KpnI and BamHI sites on the pcDNA3 (Invitrogen) expression vector in frame with a Cter Flag-tag or in fusion with a Cter YFP-Flag-tag under the endogenous or actin promoter.

### Autoradiography kinase assay.

An *in vitro* kinase activity assay was performed to assess the phosphorylation activity of EtROP2. Kinase assay reactions were performed using 1 μg of purified EtROP2 (rEtROP2, rDead, or CD). Recombinant proteins were incubated with heterologous substrates (casein or myelin basic protein [MBP]) or cell lysate at 37°C for 30 min in a kinase buffer (60 mM Tris HCl, pH 7.5, 60 mM MgCl_2_, 6 mM MnCl_2_, 0.1 M NaF, 0.3 M glycerophosphate) supplemented with 5 μM unlabeled ATP and 10 μCi [γ-32P] ATP (Perkin-Elmer Corporation, Waltham, MA, USA). After incubation, the reactions were stopped by the addition of Laemmli buffer and heat denaturation at 95°C for 5 min. Phosphorylated proteins were separated on 10% SDS-PAGE gels. Coomassie brilliant blue was used to stain the gels, which were then placed in a gel dryer (Bio-Rad Corporation, Hercules, CA, USA) and exposed to an X-ray film in an autoradiography cassette (Bio-Rad) before visualization by autoradiography.

### Protein expression and purification.

HEK 293T cells were transfected with plasmids enabling the expression of different forms of EtROP2 in frame with Flag-tag or YFP-Flag-tag (pcDNA3), using FuGENE 6 (Promega) according to the manufacturer’s instructions (26). Plasmids enabling the expression of baculovirus expressing EtROP2-GST (WT, full-length, from Met1 to Glu671) or EtROP2-Dead-GST (full-length, from Met1 to Glu671, in which the aspartate from the HxD motif was mutated into alanine, D513A) were produced by Genscript Biotech Corporation (Piscataway, NJ, USA). Each construction was cloned in the pFastBac plasmid. Recombinant proteins EtROP2-GST and EtROP2-Dead-GST were produced in Sf9 cells using the Bac-to-Bac baculovirus expression system as described in reference 64. All produced proteins were stored at −80°C in 15% glycerol final concentration.

### ADP-Glo kinase assay.

Kinase reactions were performed in white opaque, flat-bottom 384-well microplates (Optiplate, PerkinElmer) in a final volume of 6 μL, comprising the kinase reaction buffer (25 mM Tris-HCl, pH 7.5, 10 mM MgCl_2_, 1 mM EGTA, 1 mM dithiothreitol [DTT], 50 μg/mL heparin, 3 μg/mL bovine serum albumin [BSA]), tested compounds or dimethyl sulfoxide (DMSO; the final concentration of DMSO in each assay had to be 1%), recombinant proteins EtROP2-GST or EtROP2-Dead-GST (500 nM), protein substrates (MBP and casein at 520 nM) or peptide substrate (p120-TLTR at 440 nM), and 10 μM ATP (except for *K_m_*_, ATP_ determination, where a range of ATP concentrations from 0 to 100 μM was used). Plates were incubated for 30 min at 30°C before to measure the enzymatic activity using the ADP-Glo kinase assay (Promega Corporation, Madison, WI, USA) using the following protocol: (i) 6 μL of ADP-Glo reagent was added, and the plates were incubated for 50 min at room temperature; (ii) 12 μL of kinase detection reagent was added, and the plates were incubated for 60 min at room temperature. Plates were gently agitated during all incubation processes. Luminescence was measured with an EnVision multimode plate reader (PerkinElmer). All measures were performed in technical triplicates, except for the IC_50_ determination, which was done in technical quadruplicates. The data were processed using GraphPad Prism 8 software to fit a sigmoidal curve that allowed the determination of IC_50_ values. R-squared was determined as the goodness-of-fit measure to characterize how the sigmoidal curve model fit our experimental data (R-squared > 85%).

### RT-qPCR analyses.

RT-qPCR was run to monitor the parasite stages across the life cycle. Cecal RNA extraction was done with a NucleoSpin RNA kit (Macherey-Nagel) on two chickens from the first to the seventh dpi, in order to avoid individual variability. *Etama1*, *Etrop80* (21), *Etsag3*, and *Etgam56* allow the detection of sporozoites, first-generation schizonts/merozoites, second-generation schizonts/merozoites, and gametes/nonsporulated oocysts, respectively. Results were normalized using parasite housekeeping genes (*Etactin*, *Et18S*).

Nine parasite developmental stages from WT EtINRAE and five from recombinant p27700-EtROP2-YFP-Flag were purified from *in vitro* and *in vivo* experiments. Parasite purification and RNA extraction was conducted as described (21): nonsporulated oocysts (NSO), sporulated oocysts (SO), extracellular sporozoites (eSPZ), intracellular sporozoites (iSPZ), immature schizont (ImmSCHZ), mature schizont (MatSCHZ), first-generation merozoites (MI), second-generation merozoites (MII), and gametes (GAM). Briefly, intracellular sporozoites (iSPZ) and schizonts (ImmSCHZ and MatSCHZ) were recovered from infected MDBK cells at 24 hpi, 48 hpi, and 62 hpi, respectively. MII and GAM were recovered from chicken cecal mucosa 5 dpi and 6 dpi, respectively.

### Parasite transfection and selection.

We generated the EtINRAE strain expressing EtROP2 or EtROP2-Dead-tagged YFP-Flag in their Cter extension and under EtROP2 5′UTR control or under the E. tenella actin promoter by subcloning them into the pAct-Nluc vector. Recombinant parasites expressing the p27700-EtROP2-YFP-Flag, pAct-*Et*ROP2-YFP-Flag, or pAct-*Et*ROP2-Dead-YFP-Flag construction were obtained by electroporation of EtINRAE sporozoites and inoculated to chicken cloaca. Then, 7 days postinfection, the chickens were euthanized, and parasites were recovered from the ceca as previously described (65). Sporulated oocysts were then sorted by fluorescence-activated cell sorting (FACS) in order to select recombinant oocysts that were successively propagated in chickens to stabilize recombinant populations of parasites. All the processes are described in reference 66 and are summarized in Fig. S5. The parasites were propagated by oral infection (10^4^ sporulated oocysts) of 3- to 6-week-old outbred PA12 chickens and reared in a coccidia-free environment with an *ad libitum* supply of filtered water and anticoccidial- and antibiotic-free feed, following the standard protocol for oocyst purification (65).

### Western blot analysis.

A total of 1 × 10^6^ parasites from NSO, SO, eSPZ, MI, and MII stages were denatured in sample buffer, boiled for 5 min at 95°C, and resolved by SDS-PAGE. Proteins were transferred onto polyvinylidene difluoride (PVDF) membranes (Bio-Rad). The membranes were blocked by incubation in TBST (20-mMTris, 150-mM NaCl, 0.1% [vol/vol] Tween 20) containing 5% nonfat dry milk and incubated with anti-Flag primary antibody (Sigma Corporation Saint Louis, MO, USA, no. F18-04; 1:1,000). Profilin (developed in our lab; 1:200) was used as the loading control. Detection was performed with horseradish peroxidase (HRP)-secondary antibodies (Pierce-Thermo Scientific; 1:1,000).

### Immunofluorescence assay.

A total of 1 × 10^5^ HEK 293T cells were grown on a coverslip prior to transfection of 3 μg of plasmids: pcDNA-YFP-Flag, pcDNA-EtROP2-YFP-Flag, and pcDNA-EtROP2-Dead-YFP-Flag using 3 μL of FuGENE 6 (Promega, no. E2692) and 30 μL of Opti-MEM (Gibco, no. 31985062) according to the manufacturer’s recommendations. Transfected cells were cultured for 48 h, washed 3 times with phosphate-buffered saline (PBS), and fixed for 30 min with 300 μL of Antigenfix (Diapath, no. P0014). After permeabilization, phosphorylation of the p38 MAPK and JNK pathways was detected with monoclonal antibodies directed against anti-Phospho-p38 MAPK Thr^180^/Tyr^182^ (Cell Signaling, D3F9; 1:16,00), anti-phospho-c-Jun Ser^73^ (Cell Signaling, no. D47G9; 1:800), and anti-phospho-c-Jun Ser^63^ (Cell Signaling, no. E6I7P; 1:800). Staining was performed with Alexa 594-conjugated secondary antibody (Life Technologies, no. A11012). Coverslips were mounted with PermaFluor (Thermo Scientific, no. TA-030-FM) after the nucleus was stained with Hoechst stain (Invitrogen, no. H3570).

For the invasion and development assay, CLEC213 cells were infected with a multiplicity of infection (MOI) of 3 (EtINRAE mCherry, p27700-EtROP2-YFP-Flag, pAct-*Et*ROP2-YFP-Flag, and pAct-EtROP2-Dead-YFP-Flag) from 24 to 72 hpi. mCherry, YFP, and polyclonal anti-*Eimeria* spp. antibodies (1:500) were used to detect the parasites, and nuclei were stained with Hoechst stain. All images were acquired on a fluorescence microscope (Zeiss Axiovert 200 microscope) and processed with Zen 3.1 (Blue edition) software for numeration and area analysis (Fig. S6). To cope with biological variability, nine replicates were performed. Calculations are detailed as follows:

Percentage of invasion = number of infected cells/total cell number

Percentage of schizont = (immature+mature schizont)/total cell number

Schizont development (%) = (immature+mature schizont)/invaded sporozoite

### Transmission electron microscopy (TEM).

A total of 1 × 10^7^ sporozoites were fixed for 2 h with 4% paraformaldehyde in phosphate buffer (pH 7.6), washed, and centrifuged. Sporozoite pellet was included in gelatin (12%) and infused with sucrose (2.3 M). Then 90-nm ultrathin cryo-sections (Leica UCT cryo-ultramicrotome) were incubated with anti-FLAG antibody (F7425, Merck, Darmstadt, Germany) and revealed with gold-conjugated (6 nm) goat-anti-rabbit IgG (Aurion, Wageningen, Netherlands). The sections were imaged in a transmission electron microscope at 100 kV (JEOL 1011, Tokyo, Japan).

### RNA sequencing analysis.

CLEC213 cells were transfected with 10 μg of plasmids (pcDNA-eGFP-Flag or pcDNA-*Et*ROP2-YFP-Flag) as described above. After 48 h, cells were sorted by FACS and RNA extracted. RNA integrity was checked according to RNA 6000 Nano chips run on a Bioanalyzer 2100 device (Agilent Technologies, Santa Clara, CA, USA). The RNA integrity number (RIN) varies from 9 to 10.

RNA preparation was used to construct strand-specific single-end cDNA libraries according to the manufacturer’s instructions (TruSeq stranded mRNA sample prep kit, Illumina). An Illumina NextSeq 500 sequencer was used to sequence the libraries (75-bp single-end reads). The transcriptome sequencing (RNA-seq) analysis was performed with the Sequana framework (67). We used the RNA-seq pipeline (v0.9.16), which is available online (https://github.com/sequana/sequana_rnaseq). Reads were trimmed from adapters using Cutadapt (v2.10) and then mapped to the Gallus gallus genome assembly from Ensembl using STAR (v2.7.3a). FeatureCounts (v2.0.0) was used to produce the count matrix, assigning reads to features using annotation GRCg6a with strand-specificity information. Quality control statistics were summarized using MultiQC (v1.8). Statistical analysis of the count matrix was performed to identify differentially regulated genes, comparing cell expressed a parasite protein (EtROP2) with empty vector samples. Clustering of transcriptomic profiles was assessed using a principal-component analysis (PCA). Differential expression testing was conducted using DESeq2 library (v1.24.0) scripts based on SARTools (v1.7.0) (68), indicating the significance (Benjamini-Hochberg-adjusted *P* values, false-discovery rate [FDR] < 0.05) and the effect size (fold change) for each comparison.

### Histological analysis.

Outbred PA12 chickens aged 4 to 6 weeks were infected orally with sporulated oocysts (2.5 × 10^5^ oocysts for necropsies at 1 to 4 dpi and 10^4^ oocysts for necropsies at 5 to 7 dpi). Ceca from two infected animals with EtINRAE or pAct-EtROP2-YFP-Flag were fixed in formalin from the first to the seventh day p.i. and embedded in paraffin. Serial longitudinal sections of 4 μm thick were made and stained with hemalun eosin saffron.

### Data availability.

The data sets produced in this study are available in the GEO repository database, under accession number GSE205391.
