# Supplementary material for: Overexpression of Eimeria tenella Rhoptry Kinase 2 Induces Early Production of Schizonts
Source: Microbiol Spectr. 2023 Jun 1;11(4):e00137-23. doi: 10.1128/spectrum.00137-23 (PMC10434272; doi:10.1128/spectrum.00137-23)
Supplement: Supplemental file 1 — Supplemental material. Download spectrum.00137-23-s0001.docx, DOCX file, 0.01 MB [file spectrum.00137-23-s0001.docx]

**Supplementary data**

**Figure S1: Characterization of recombinant parasite strains.**

A. Immunogold revelation of the Flag of the p27700-EtROP2-YFP-Flag, pAct-*Et*ROP2-YFP-Flag and pAct-EtROP2-Dead-YFP-Flag strains was performed by TEM and confirmed the rhoptry localization of the recombinant protein. Immunofluorescence assay of CLEC213 cells infected by sporozoites from each strain, confirmed the YFP signal in the apical tip of the sporozoites (nuclei stained in DAPI, blue; sporozoites revealed by polyclonal anti-*Eimeria* spp antibodies and GAR-594). B. Transcription analysis on p27700-*Et*ROP2-YFP-Flag (green), pAct-*Et*ROP2-YFP-Flag strain (red), pAct-*Et*ROP2-Dead-YFP-Flag strain (grey), sporulated oocysts, with primers allowing the *Etrop2* and *yfp* amplification. The overexpression of the active form of the kinase in pAct parasites is confirmed but not for the inactive kinase in Dead parasites. Ordinary one-way ANOVA was performed using GraphPad Prism and Tukey’s test was used to correct for multiple comparison. **** p-value <0.0001.

**Figure S2: Histological analysis of infected chickens at day 3 post-infection.**

Caeca were collected from infected chickens with WT *Et*INRAE-YFP (A) or pAct-*Et*ROP2-YFP-Flag (B) strains. The Hemalun-eosin saffron coloration allows the visualization of the parasites in the mucosa. For the WT *Et*INRAE-YFP strain, immature schizonts (filled arrowheads) are visualized inside the lamina propria while, for pAct-*Et*ROP2-YFP-Flag strain, immature and mature (open arrowheads) schizonts are visualized.

**Figure S3: Invasion assay analysis.**

Invasion was measured on CLEC213 infected with four different strains, *Et*INRAE-YFP strain (black), p27700-*Et*ROP2-YFP-FLAG (green), pAct-*Et*ROP2-YFP-Flag strain (red), pAct-*Et*ROP2-Dead-YFP-Flag strain (grey), at 2 h.p.i. with no differences between the strains. Results are expressed as percentage of invasion ± SD. Nine replicates were performed in order to cope with biological variability. Ordinary one-way ANOVA was performed using GraphPad Prism.

**Figure S4: Volcano-plot of the dataset.**

The volcano-plot represents the data for the comparison between pcDNA-*Et*ROP2-YFP-Flag and pcDNA-eGFP-Flag where significantly differentially expressed features are highlighted in red. Genes from p38 MAPK pathway overexpressed in pcDNA-*Et*ROP2-YFP-Flag in comparison to pcDNA-eGFP-Flag are highlighted in green. A volcano plot represents the log of the adjusted P-values as a function of the log ratio of differential expression.

**Figure S5: Production and selection of recombinant *E. tenella* strains.**

Recombinant strains were obtained after plasmid electroporation in freshly excysted sporozoites and inoculation to chicken cloaca. Seven days post-infection, recovered oocysts were incubated for sporulation and recombinant parasites were enriched by fluorescence-activated cell sorting (FACS). Selected recombinant oocysts were propagated in chickens by oral infection, to stabilize recombinant populations of parasites. Figure S5 was created by BioRender.com (agreement number UO24JVHCZE, for Microbiology Spectrum journal).

**Figure S6: Determination of immature and mature schizont area.**

Area of immature (open arrowheads) and mature (filled arrowheads) schizonts were determined by drawing the outline as indicated by dotted lines. Images were acquired on a fluorescence microscope and processed with Zen3.1 software. This picture represents the immunofluorescence assay realized for the wild-type parasite strain at 72 h.p.i.

**Table S1: RNA-seq transcriptome analysis of CLEC213 cells transfected with *Et*ROP2.**

The datasets produced in this study are available in the GEO repository database, under the accession number GSE205391.

**Table S2: KEGG pathway identified for differentially expressed genes in *Et*ROP2 transfected CLEC213 cells.**

**Table S3: List of primers used for plasmids construction and used in real time qRT-PCR analysis**
