# Supplementary figures and images for: Overexpression of Eimeria tenella Rhoptry Kinase 2 Induces Early Production of Schizonts
Source: Microbiol Spectr. 2023 Jun 1;11(4):e00137-23. doi: 10.1128/spectrum.00137-23 (PMC10434272; doi:10.1128/spectrum.00137-23)

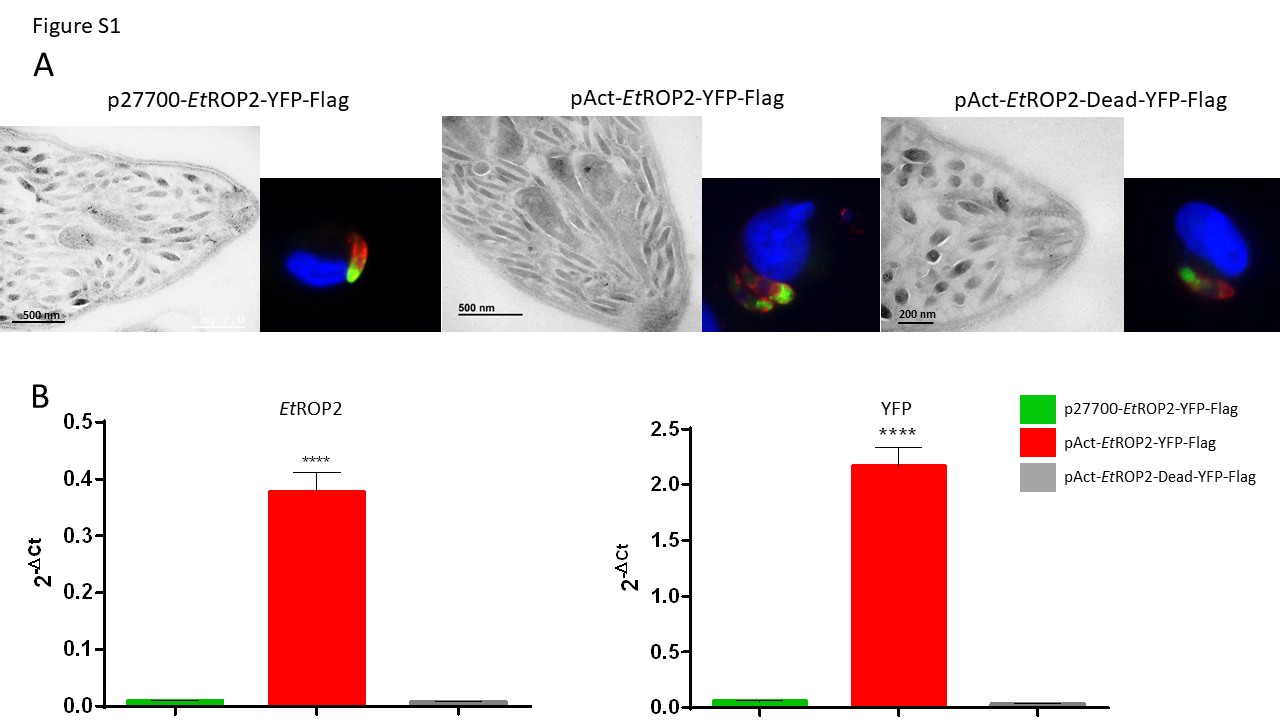

Supplement: Supplemental file 3 — Supplemental material. Download spectrum.00137-23-s0003.tif, TIF file, 0.3 MB [file spectrum.00137-23-s0003.tif]

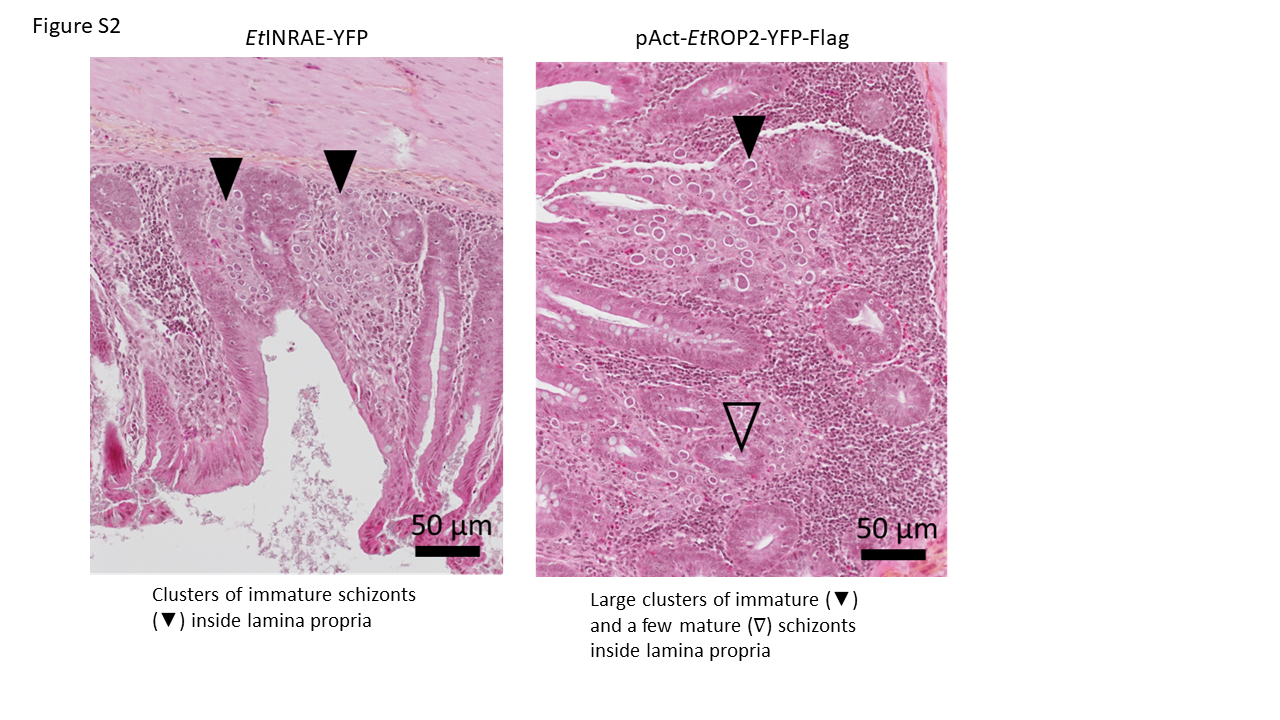

Supplement: Supplemental file 4 — Supplemental material. Download spectrum.00137-23-s0004.tif, TIF file, 1.1 MB [file spectrum.00137-23-s0004.tif]

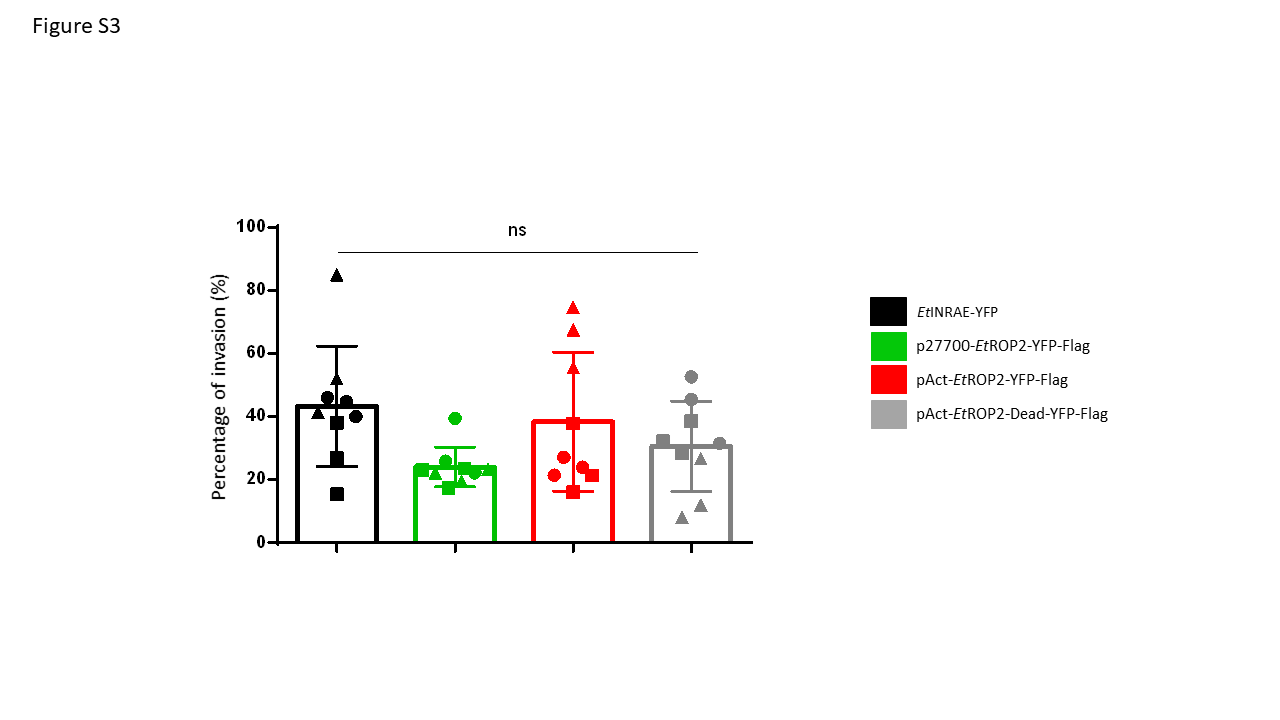

Supplement: Supplemental file 5 — Supplemental material. Download spectrum.00137-23-s0005.tif, TIF file, 0.07 MB [file spectrum.00137-23-s0005.tif]

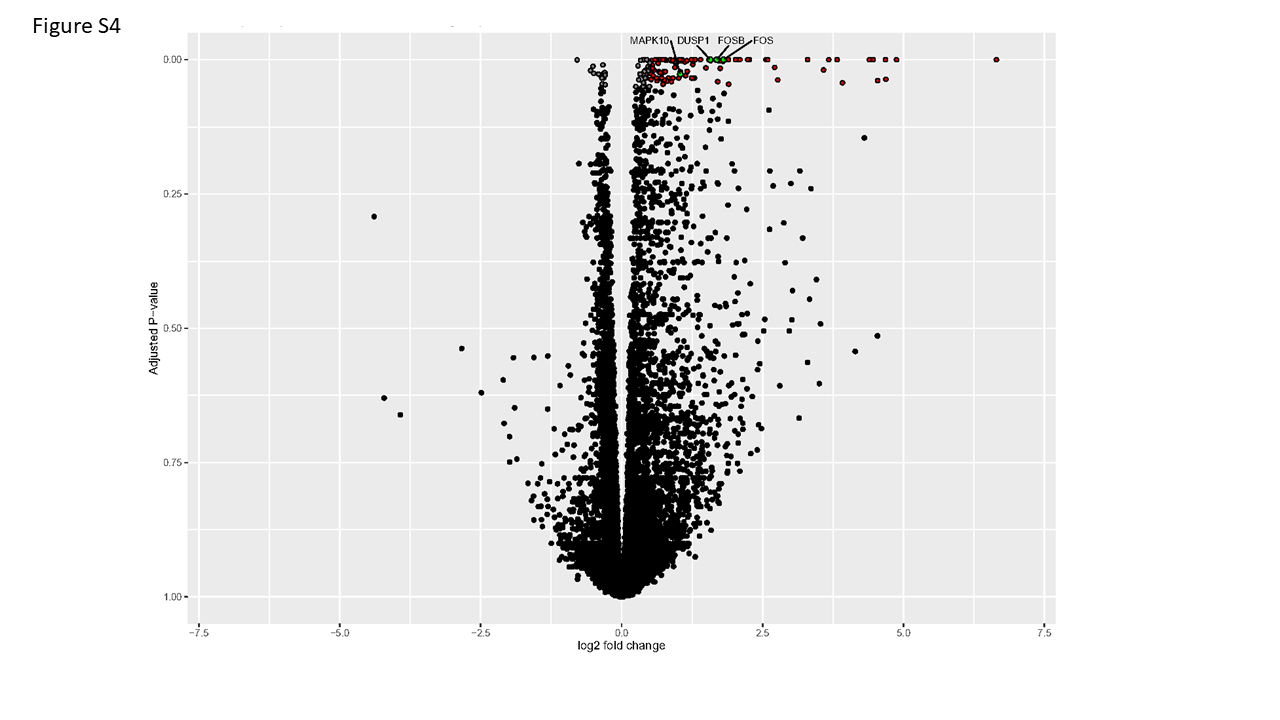

Supplement: Supplemental file 6 — Supplemental material. Download spectrum.00137-23-s0006.tif, TIF file, 0.2 MB [file spectrum.00137-23-s0006.tif]

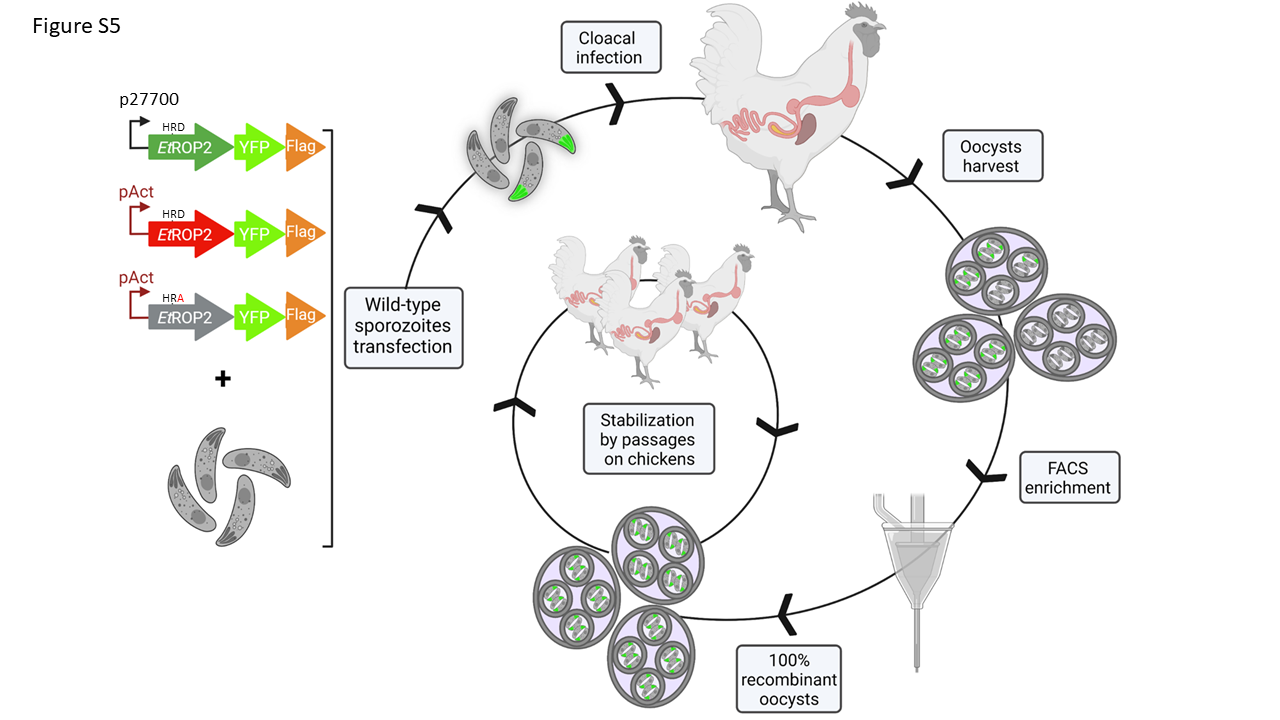

Supplement: Supplemental file 7 — Supplemental material. Download spectrum.00137-23-s0007.tif, TIF file, 0.3 MB [file spectrum.00137-23-s0007.tif]

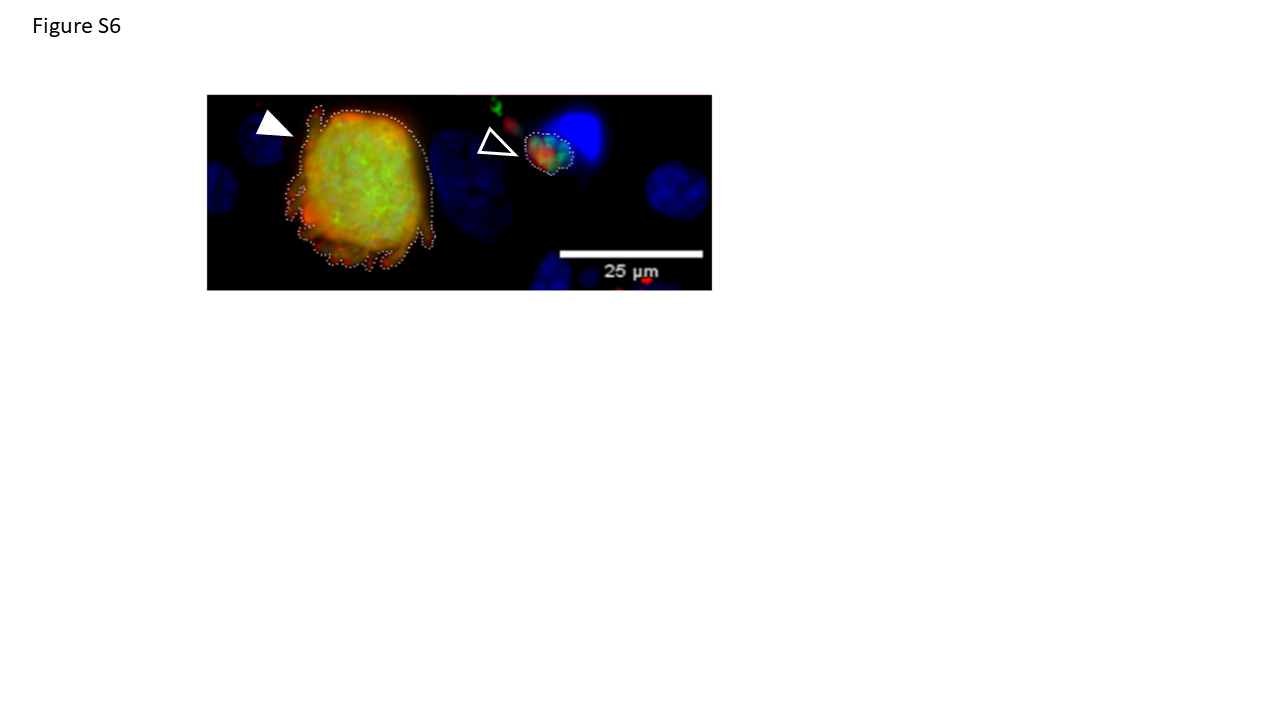

Supplement: Supplemental file 8 — Supplemental material. Download spectrum.00137-23-s0008.tif, TIF file, 0.1 MB [file spectrum.00137-23-s0008.tif]
